# Use of Nanostructure-Initiator Mass Spectrometry to Deduce Selectivity of Reaction in Glycoside Hydrolases

**DOI:** 10.3389/fbioe.2015.00165

**Published:** 2015-10-27

**Authors:** Kai Deng, Taichi E. Takasuka, Christopher M. Bianchetti, Lai F. Bergeman, Paul D. Adams, Trent R. Northen, Brian G. Fox

**Affiliations:** ^1^US Department of Energy Joint BioEnergy Institute, Emeryville, CA, USA; ^2^Sandia National Laboratories, Livermore, CA, USA; ^3^US Department of Energy Great Lakes Bioenergy Research Center, Madison, WI, USA; ^4^Department of Chemistry, University of Wisconsin-Oshkosh, Oshkosh, WI, USA; ^5^Lawrence Berkeley National Laboratory, Berkeley, CA, USA; ^6^Department of Bioengineering, University of California Berkeley, Berkeley, CA, USA; ^7^Department of Biochemistry, University of Wisconsin-Madison, Madison, WI, USA

**Keywords:** cellulase, assay, kinetics, Nimzyme, mass spectrometry, protein engineering, biofuels

## Abstract

Chemically synthesized nanostructure-initiator mass spectrometry (NIMS) probes derivatized with tetrasaccharides were used to study the reactivity of representative *Clostridium thermocellum* β-glucosidase, endoglucanases, and cellobiohydrolase. Diagnostic patterns for reactions of these different classes of enzymes were observed. Results show sequential removal of glucose by the β-glucosidase and a progressive increase in specificity of reaction from endoglucanases to cellobiohydrolase. Time-dependent reactions of these polysaccharide-selective enzymes were modeled by numerical integration, which provides a quantitative basis to make functional distinctions among a continuum of naturally evolved catalytic properties. Consequently, our method, which combines automated protein translation with high-sensitivity and time-dependent detection of multiple products, provides a new approach to annotate glycoside hydrolase phylogenetic trees with functional measurements.

## Introduction

The enzymatic hydrolysis of plant cell wall material is a formidable task because of the complexity of the plant cell wall (Himmel et al., 2007). In most currently deployed cellulosic ethanol plants, enzyme cocktails containing multiple classes of polysaccharide-degrading enzymes are used to hydrolyze plant biomass into fermentable sugars. Understanding the function, synergy, and stability of enzymes is thus of paramount importance in biofuels production.

Polysaccharide-degrading enzymes are classified into families in the carbohydrate active enzyme (CAZy) database (Henrissat and Davies, 1997; Cantarel et al., 2009; Levasseur et al., 2013), including glycoside hydrolases (GHs), pectic lyases (PLs), carbohydrate esterases (CEs), and others. Only a small fraction of the enzymes included in CAZy have a function assigned by biochemical analyses. One root of this limitation arises from difficulties in succeeding with heterologous expression of enzymes after selection from phylogenetic trees (Watson et al., 2007; Fox et al., 2008; Markley et al., 2009; Nair et al., 2009; Pieper et al., 2013). As an option to address this limitation, we (Takasuka et al., 2014; Bianchetti et al., 2015) and others (Beebe et al., 2011, 2014; Madono et al., 2011; Hirano et al., 2013, 2015; Makino et al., 2014) have found that wheat germ cell-free protein translation can be used as an effective expression platform to make functional assignments of enzyme function.

Another limitation arises from experimental complications of carrying out high-throughput multisubstrate assays to screen for enzyme function (Gerlt et al., 2011). A breadth of assay methods have been developed for GHs, including use of soluble and insoluble chromogenic and/or fluorogenic substrates, HPLC, and others (Sharrock, 1988; Decker et al., 2003; Chundawat et al., 2008; Bansal et al., 2009; Dowe, 2009; Dashtban et al., 2010; Selig et al., 2011; Eklof et al., 2012; Horn et al., 2012; Kosik et al., 2012; McCleary et al., 2012; Pena et al., 2012; Whitehead et al., 2012; Wischmann et al., 2012). Each of these approaches has intrinsic advantages, but can suffer in sensitivity, complexity of analysis, throughput time, and volumes of reagents and enzyme needed. In comparison, nanostructure-initiator mass spectrometry (NIMS) offers high sensitivity, simplicity of detection of products derived from biomass hydrolysis, microliters or smaller volumes for reaction, and options for automation (Northen et al., 2008; Deng et al., 2012; de Rond et al., 2013; Heins et al., 2014). Recently, we used oxime-NIMS and numerical integration methods to provide time-dependent, quantitative characterization of reducing sugars released by individual enzymes in reactions with pretreated biomass (Deng et al., 2014).

Here, we report a new use of NIMS to provide quantitative analysis of time-dependent reactions of cellulases. The enzymes selected for this study were from *Clostridium thermocellum*, a Gram-positive anaerobe with high cellulolytic capacity (Ding et al., 2008; Fontes and Gilbert, 2010; Smith and Bayer, 2013). The *C. thermocellum* genome encodes ~130 CAZyme domains and ~90 carbohydrate-binding module (CBM) domains (Feinberg et al., 2011). The majority of CAZyme domains also possess dockerin domains, which serve to recruit these enzymes into the cellulosome via dockerin–cohesin interactions (Ding et al., 2008; Smith and Bayer, 2013). The specific gene regulatory and protein secretory patterns of this model consolidated bioprocessing organism have also been well described (Brown et al., 2007; Gold and Martin, 2007; Roberts et al., 2010; Feinberg et al., 2011; Raman et al., 2011; Riederer et al., 2011), and many of the enzymes have been characterized. Given this state of knowledge, individual enzymes from *C. thermocellum* have proven useful for the development and testing of new approaches for assignment of GH function.

In this work, we have used chemically synthesized tetrasaccharide-NIMS probes to study the reactivity of some cellulases from *C. thermocellum*. Patterns of reactivity identified by using the tetrasaccharide-NIMS probes provide a diagnostic approach to assess reaction specificity and also provide comparative apparent rate information. Our results show diagnostic patterns for reactions of a β-glucosidase, relaxed but varied specificity of several endoglucanases and high specificity of a cellobiohydrolase with the model substrate. Time-dependent reactions of these polysaccharide-selective enzymes were modeled by numerical integration, which provides a quantitative basis to make functional distinctions among a continuum of naturally evolved reactive properties. Consequently, this method, which combines high-sensitivity detection of multiple products with quantitative numerical analysis of their time-dependent formation, provides a new approach to enhance the annotation of GH phylogenetic trees with functional measurements.

## Materials and Methods

### Enzyme Preparation

Methods for cloning, cell-free translation, and purification of the enzymes studied have been reported elsewhere (Takasuka et al., 2014). Briefly, enzymes were cloned by PCR amplification of catalytic domains as indicated by the first and last codons indicated in Table 1. Cloned genes were transferred into an optimized wheat germ cell-free translation plasmid pEU-HSCB (Beebe et al., 2011; Takasuka et al., 2014), which is also available from the NIH Protein Structure Initiative Materials Repository (http://psimr.asu.edu/). Enzymes were prepared by cell-free translation using either bilayer or dialysis methods (Beebe et al., 2011, 2014; Makino et al., 2014), and active enzymes were identified (Takasuka et al., 2014). The enzymes listed in Table 1 were also cloned by PCR into the *Escherichia coli* expression vector pEC_CBM3a to create enzyme_CBM3a fusion proteins, CelAcc_CBM3a. The vector pEC_CBM3a is a derivative of pEU_HSBC_CBM3a (Takasuka et al., 2014) that yields fusion proteins having an N-terminal enzyme catalytic domain fused by an ~40 aa linker sequence to the CBM3a domain from Cthe_3077. A stop codon was added to the PCR primer used to amplify the 3′ end of the BglA gene so that no fusion to CBM3a was produced from pEU_HSBC_CBM3a. As needed, protein coding sequences were transferred between pEU and pEC vectors by use of FlexiVector cloning (Blommel et al., 2009). Methods for PCR amplification, capture and sequence verification of protein coding sequences, and transformation into *E. coli* 10G competent cells (Lucigen, Middleton, WI, USA) for DNA manipulations and *E. coli* B834 for protein expression were as previously reported (Takasuka et al., 2014). Additional details of the properties and methods for the use of pEU and pVP are described elsewhere (Aceti et al., 2015).

**Table 1:** ***Clostridium thermocellum* enzymes studied in this work**.

Gene locus	Name	CAZy family	First codon^a^	Last codon^b^	Function^c^	CMX class^d^	Reference
Cthe_0212	BglA	GH1	1	448	Exo-β-glucosidase		Grabnitz et al. (1991)
Cthe_0269	CelA	GH8	34	368	Endo-β-1,4-glucanase	CX	1CEM; Alzari et al. (1996)
Cthe_0040	CelI	GH9	28	887	Endo-β-1,4-glucanase	C	2XFG
Cthe_0797	CelE	GH5	36	388	Cellulase, xylanase, mannanase	CMX	Deng et al. (2014, Takasuka)
Cthe_0578	CelR	GH9	27	640	Endo-β-1,4-glucanase; cellotetraohydrolase	C	Zverlov et al. (2005)
Cthe_0405	CelL	GH5	31	430	–	CX	Deng et al. (2014)
Cthe_0412	CelK	GH9	28	809	Cellobiohydrolase	C	Kataeva et al. (1999)
Cthe_3077^e^	CipA	CBM3a	323	523	Cellulose-binding module		Kataeva et al. (1999)

*^a^First codon of the indicated gene locus that was included in the PCR primer design (Takasuka et al., 2014)*.

*^b^Last codon of the indicated gene locus that was included in the PCR primer design*.

*^c^Function assigned from annotation as defined in CAZy (Cantarel et al., 2009), from experimental evidence cited in the table, or a combination of both*.

*^d^Representation of the breadth of substrate specificity for each enzyme (Deng et al., 2014). The CMX classification indicates that CelE can hydrolyze cellulose, xylan, or mannan; CX indicates that CelA and CelL can hydrolyze cellulose and xylan, while CelI, CelR, and CelK can only hydrolyze cellulose. This classification derives from reactions with pure polysaccharides and pretreated biomass (Deng et al., 2014; Takasuka et al., 2014)*.

*^e^CBM3a was subcloned from the scaffoldin CipA gene*.

### Synthesis of Cellotetraose-NIMS Substrate

**Figure 1:**
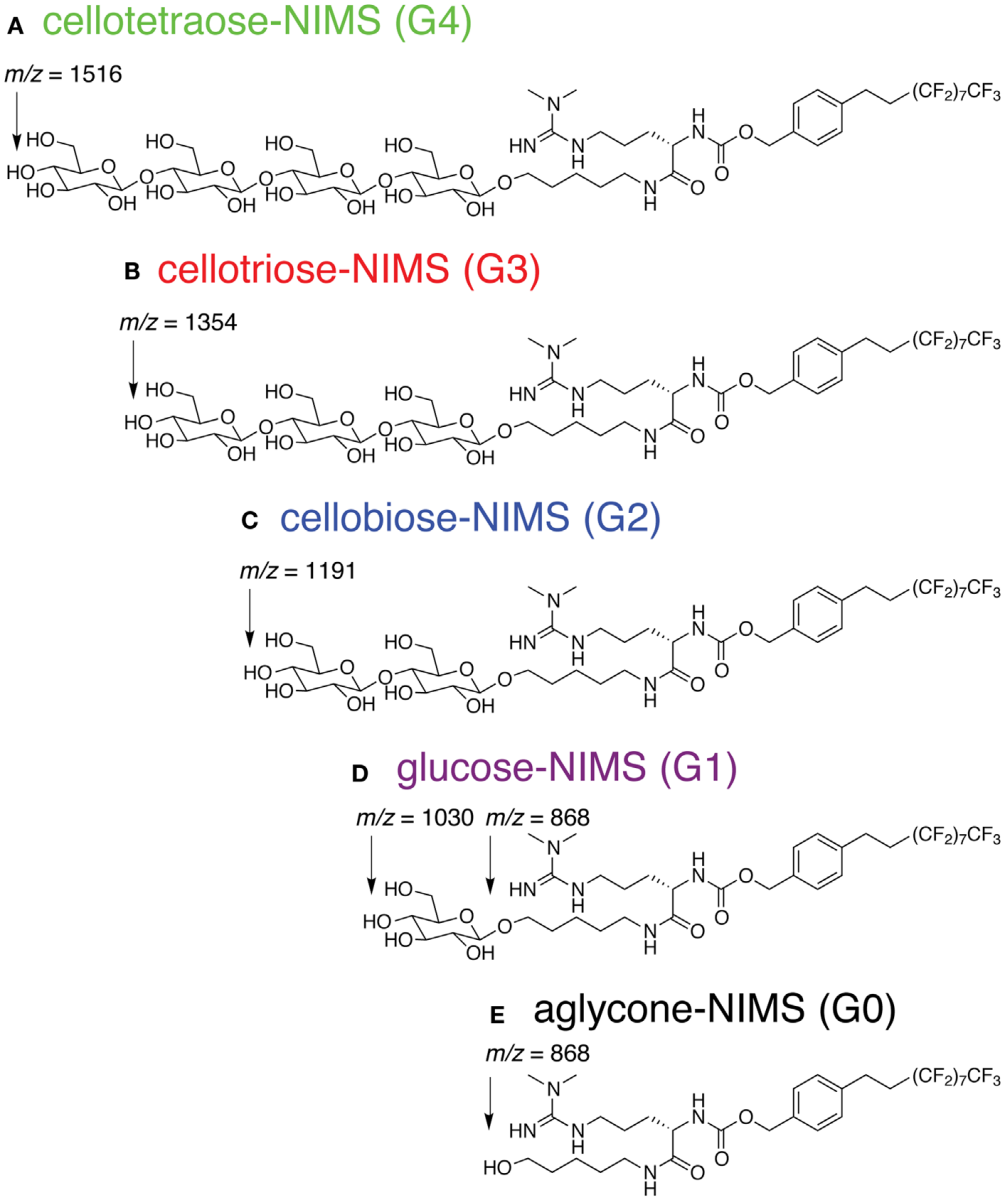
**Structure of cellotetraose-NIMS and *m/z* values for products obtained from hydrolysis at the indicated anomeric position**. **(A)** cellotetraose-NIMS; **(B)** cellotriose-NIMS; **(C)** cellobiose-NIMS; **(D)** glucose-NIMS; **(E)** aglycone-NIMS.

The cellotetraose-NIMS substrate (Figure 1A) is an amphiphilic molecule that has a sugar head group coupled to a perfluorinated (F17) tag. The detailed synthetic procedure has been reported previously (Deng et al., 2012).

### Enzyme Reactions

An enzyme reaction consisted of 10 μL of 50 mM phosphate, pH 6.0, supplemented with 1 μL of 1 mM cellotetraose-F17 dissolved in water. An aliquot of each enzyme preparation (containing 1–10 ng of enzyme) was added to initiate the reaction and the resulting mixture was incubated at 37°C. At times of 5, 10, 20, 40, 80, and 120 min, 0.2 μL of the reaction mixture was withdrawn for analysis.

### Mass Spectrometry

**Figure 2:**
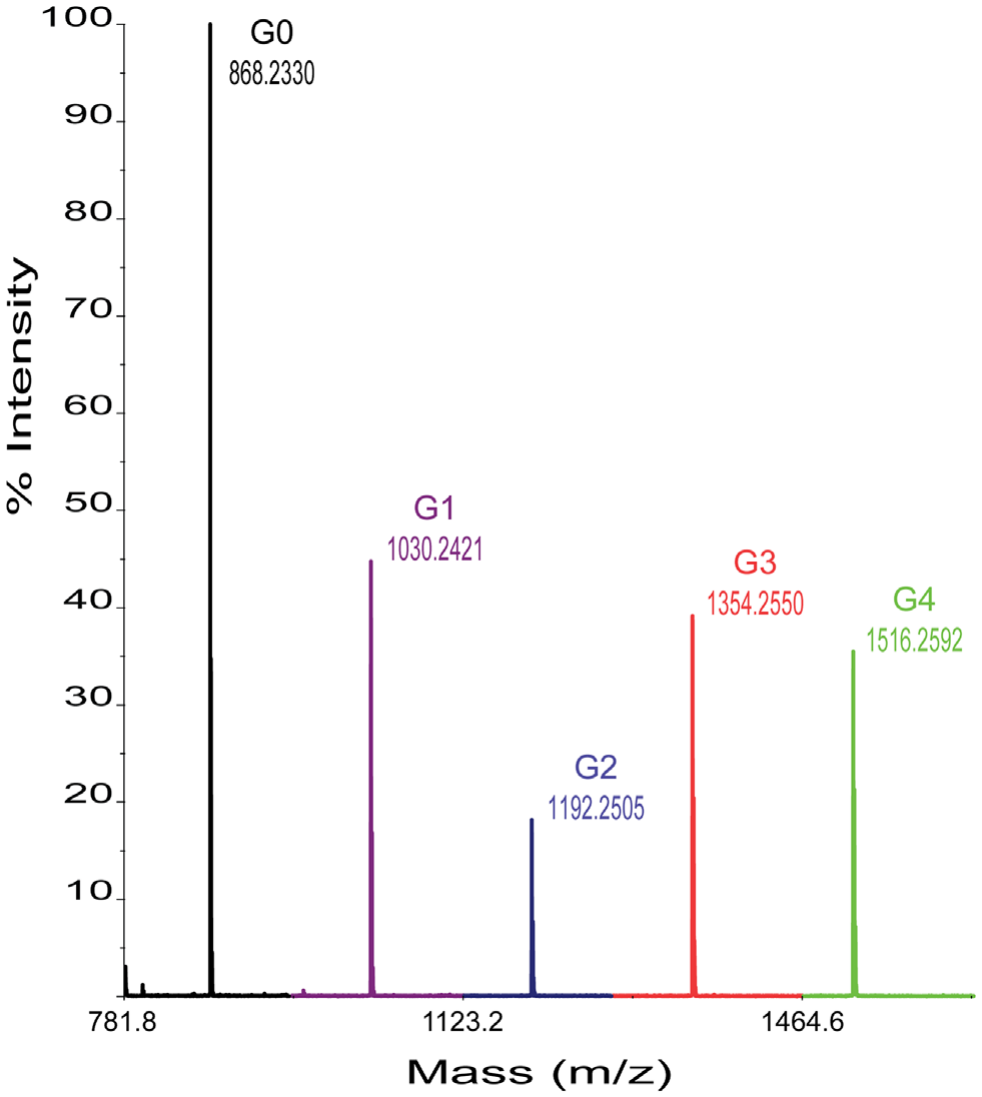
**Representative mass spectrum obtained from enzyme hydrolysis of cellotetraose-NIMS**. Mass peaks corresponding to cellotetraose-NIMS (green), cellotriose-NIMS (red), cellobiose-NIMS (blue), glucose-NIMS (purple), and aglycone-NIMS (black) are indicated. The products shown are from reaction of BglA.

In each case, 0.2 μL per reaction sample was spotted onto the NIMS surface and removed after an incubation of ~30 s. A grid drawn manually on the NIMS chip using a diamond-tip scribe helped with spotting and identification of sample spots in the spectrometer. Chips were loaded using a modified standard MALDI plate. NIMS was performed on a 4800 MALDI TOF/TOF mass analyzer from AB Sciex (Foster City, CA, USA). In each case, signal intensities were identified for the ions of the cellotetraose substrate and, when present, each product shown in Figure 1. For each assay, ~1000 laser shots were collected. Enzyme activities were determined by measuring the intensity ratios of each product over the intensity total of ions of for the cellotetraose-, cellotriose-, cellobiose-, glucose-, and aglycone-NIMS (Figure 2).

### Kinetic Analyses

**Figure 3:**
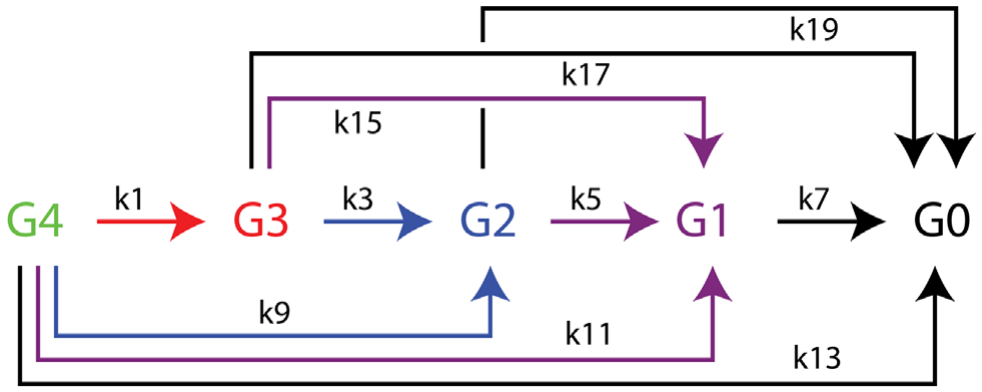
**Kinetic scheme for the enzymatic hydrolysis of cellotetraose-NIMS accounting for all products detected**. Apparent rate constants determined from numerical simulations of time dependence of enzyme reactions using differential equations 1–10 from the section “Materials and Methods” are found in Table 2. Cellotetraose-NIMS, green; cellotriose-NIMS, red; cellobiose-NIMS, blue; glucose, purple; aglycone-NIMS, black.

The time dependence of hydrolysis of the tetrasaccharide-NIMS was analyzed by non-linear global optimization of differential equations accounting for the appearance and decay of products (Deng et al., 2014) using Mathematica routine NDSolve and the Nelder-Mead simplex method for constrained minimization (Nelder and Mead, 1965). The differential equations corresponding to the kinetic scheme of Figure 3 are as follows:

(1) y[1]=cellotetraose-NIMS

(2) y[2]=cellotriose-NIMS

(3) y[3]=cellobiose-NIMS

(4) y[4]=glucose-NIMS

(5) y[5]=aglycone-NIMS

(6) dy[1]/d[t]=−(k1+k9+k11+k13 ) y[1] [t]

(7) dy[2]/d[t]=(k1) y[1] [t]−(k3+k15+k17) y[2] [t]

(8) dy[3]/d[t]=(k9) y[1] [t]+(k3) y[2] [t]−(k5+k19) y[3] [t]

(9) dy[4]/d[t]=(k11) y[1] [t]+(k15) y[2] [t]+(k5) y[3] [t]−(k7) y[4] [t]

(10) dy[5]/d[t]=(k13) y[1] [t]+(k17) y[2] [t]+(k19) y[3] [t]+(k7) y[4] [t]

Initial guesses for apparent rate constants were made by visual inspection of the match between the results of single NDSolve calculations and the experimental data. This process was continued in an iterative way until a set of initial apparent rates that adequately matched the experimental data was obtained. Successive rounds of least squares parameter optimization with adjustment of parameter constraints were carried out until the sum of the squares difference between calculated and experimental values reached a minimum and no parameter was artificially constrained.

## Results and Discussion

### Enzymes Chosen for Study

*Clostridium thermocellum* enzymes were chosen for this study based on previous transcriptomic and proteomic results (Gold and Martin, 2007; Raman et al., 2011; Riederer et al., 2011) and other biochemical and structural results (Table 1). Genes encoding these enzymes were expressed using wheat germ cell-free protein synthesis and the translated proteins were assayed using fluorogenic substrates (Takasuka et al., 2014); among the synthesized enzymes, 13 reacted with MUG or MUC, 11 reacted with MUX or MUX2, and 5 reacted with other diagnostic fluorogenic substrates. Reactions of these enzymes with ionic liquid pretreated switchgrass (IL-SG) have been published (Deng et al., 2014). Enzymes from cell-free translation reactions that showed promising characteristics were produced by expression in *E. coli* and purified for use in the studies described here.

### Cellotetraose-NIMS Substrate

Figure 1 shows the structure of cellotetraose-NIMS and the products that can be formed by various GH reactions. In the synthesized probe, the tetra-saccharide is linked to the NIMS probe by a potentially hydrolyzable anomeric linkage. Synthesis of the NIMS probe and the tetra-saccharide derivatives are summarized in Materials and Methods (Deng et al., 2012; de Rond et al., 2013). The guanidium group on the NIMS probe provides improved ionization properties in the mass spectrometry experiment, while the perfluorinated portion of the NIMS probe provides hydrophobic anchoring of the molecule into the NIMS surface. Enzyme-catalyzed hydrolysis of the anomeric linkages give rise to a cascade of potential products retained on the NIMS surface. Reactions of GHs can progressively remove single glucose units or carry out other reactions that remove cellobiose, cellotriose, or cellotetraose.

### Kinetic Scheme

Figure 2 shows a representative mass spectrum obtained after partial reaction with BglA (Cthe_0212), a β-glucosidase. At the selected time point in the reaction (120 min), the cellotetraose-NIMS probe (G4, green) has been partially converted into a mixture of cellotriose (G3, red), cellobiose (G2, blue), glucose (G1, purple), and aglycone (G0, black) derivatives of the NIMS probe. Figure 3 shows a kinetic scheme that accounts for the potential products shown in Figure 1. The scheme accounts for release of one or more glucose units from the cellotetraose-NIMS probe (G4) and its successive products. Time course profiles provide the fundamental data used in this work for numerical analysis of enzyme hydrolysis reactions.

### β-Glucosidase BglA Reaction

The nucleotide sequence of BglA (Grabnitz et al., 1991) was published before the genome sequence and annotated to be a β-glucosidase from the GH1 family (Cantarel et al., 2009). The Cthe_02012 gene does not encode a signal peptide, so the entire gene was cloned for the studies described here. Beyond our characterization of the reaction of BglA with IL-SG (Deng et al., 2014), no other functional studies have been reported for this enzyme.

**Figure 4:**
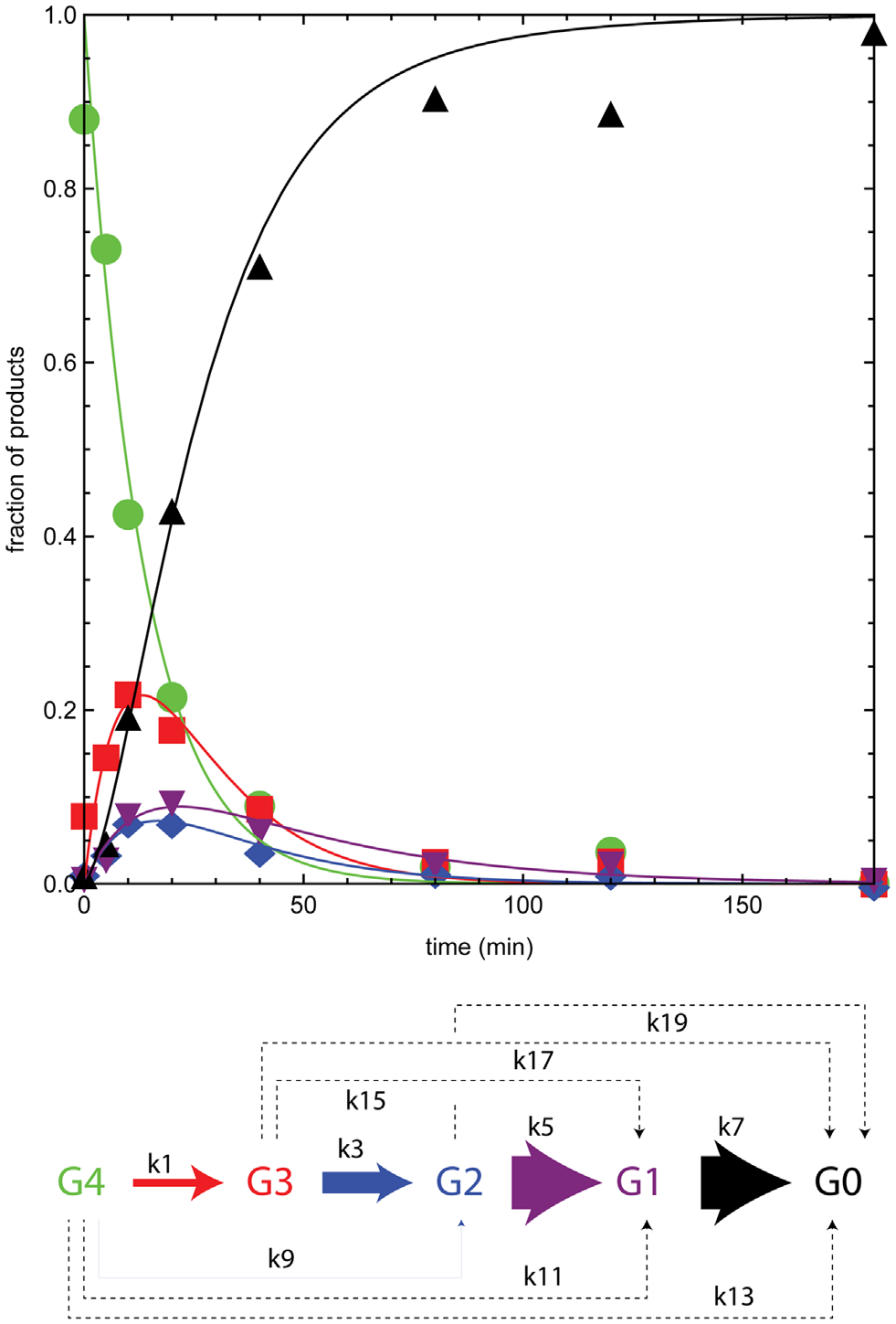
**Numerical analysis of the time course for reaction of BglA with cellotetraose-NIMS**. Products are cellotetraose-NIMS (green), cellotriose-NIMS (red), cellobiose-NIMS (blue), glucose- NIMS (purple), and aglycone-NIMS (black). Relative magnitude of the apparent rates shown in Table 2 indicated by width of arrows in the modified kinetic scheme. A dashed line indicates that the apparent rate was zero.

Figure 4 shows the time course for reaction of BglA with cellotetraose-NIMS. The plotted proportions of the different products come from time series of mass spectra like those shown in Figure 2. The solid colored lines are results of simulations of the concentration of individual products based on the kinetic scheme of Figure 3 and the differential equations shown in the section “Materials and Methods.” The apparent rate constants provided by the numerical simulation are given in Table 2, and a pictorial representation of the relative magnitudes of the apparent rate constants is also given in Figure 4. In the time course of the BglA reaction, cellotetraose-NIMS (green circles) was converted to a succession of intermediates by hydrolysis of a single glucose from the position most distal to the NIMS probe. This pattern of reactivity is as expected for the reaction of an exo-β-glucosidase with an oligosaccharide. Thus, cellotriose-NIMS (red squares) accumulated was subsequently converted to cellobiose-NIMS (purple down triangles), to glucose-NIMS (blue diamonds), and ultimately to aglycone-NIMS (black up triangles).

**Table 2:** **Apparent rate constants (min^−^^1^) from numerical integration of time course reactions with cellotetraose-NIMS**.

Rate	BglA	CelL	CelR	CelE	CelA	CelI	CelK
k1	0.05320	0.000	0.001	0.001	0.000001	0.0002	0.0000
k3	0.14805	0.0002	0.0000	0.0000	0.0000	0.0002	0.0000
k5	0.36803	0.0000	0.0000	0.0000	0.0000	0.0000	0.0000
k7	0.36803	0.0000	0.0000	0.0000	0.0000	0.0000	0.0000
k9	0.00047	0.0490	0.0170	0.0092	0.0004	0.0020	0.0439
k11	0.00006	0.0038	0.0453	0.0390	0.0116	0.0479	0.0000
k13	0.00006	0.0000	0.0001	0.0000	0.0000	0.0000	0.0000
k15	0.00006	0.0000	0.0000	0.0000	0.0010	0.0000	0.0004
k17	0.00006	0.0000	0.0000	0.0000	0.0000	0.0000	0.0000
k19	0.00006	0.0000	0.0000	0.0000	0.0000	0.0000	0.0001

*Rates for individual enzymes are color coded with smallest values in blue and larger values in red*.

There are several features of the BglA reaction and simulation that warrant attention. The apparent rates k1, k3, k5, and k7, which correspond to successive removal of single glucose groups, dominate the numerical solution (Table 2; Figure 4). Under the reaction conditions used, BglA was able to completely convert cellotetraose-NIMS to aglycone-NIMS. It is also noteworthy that shortening the oligosaccharide chain led to an enhancement in the rate of hydrolysis, with reactions k5 (converting cellobiose-NIMS to glucose-NIMS) and k7 (converting glucose-NIMS to aglycone-NIMS) being fastest. Other apparent rates corresponding to side reactions for removal of cellobiose or larger oligosaccharides (e.g., k9 for removal of cellobiose from cellotetraose-NIMS) were less than 1/100th of the value observed for k1, the smallest of the central reactions. These simulation results are consistent with the assigned function of BglA as a β-glucosidase. Indeed, prior oxime-NIMS studies of the reaction of BglA with IL-SG revealed that glucose was the only product released from the biomass substrate (Deng et al., 2014). In the following paragraphs, these diagnostic behaviors of a beta-glucosidase are contrasted with two other classes of GHs, including five phylogenetically diverse endoglucanases and one cellobiohydrolase.

### Endoglucanase and Cellobiohydrolase Reactions

**Figure 5:**
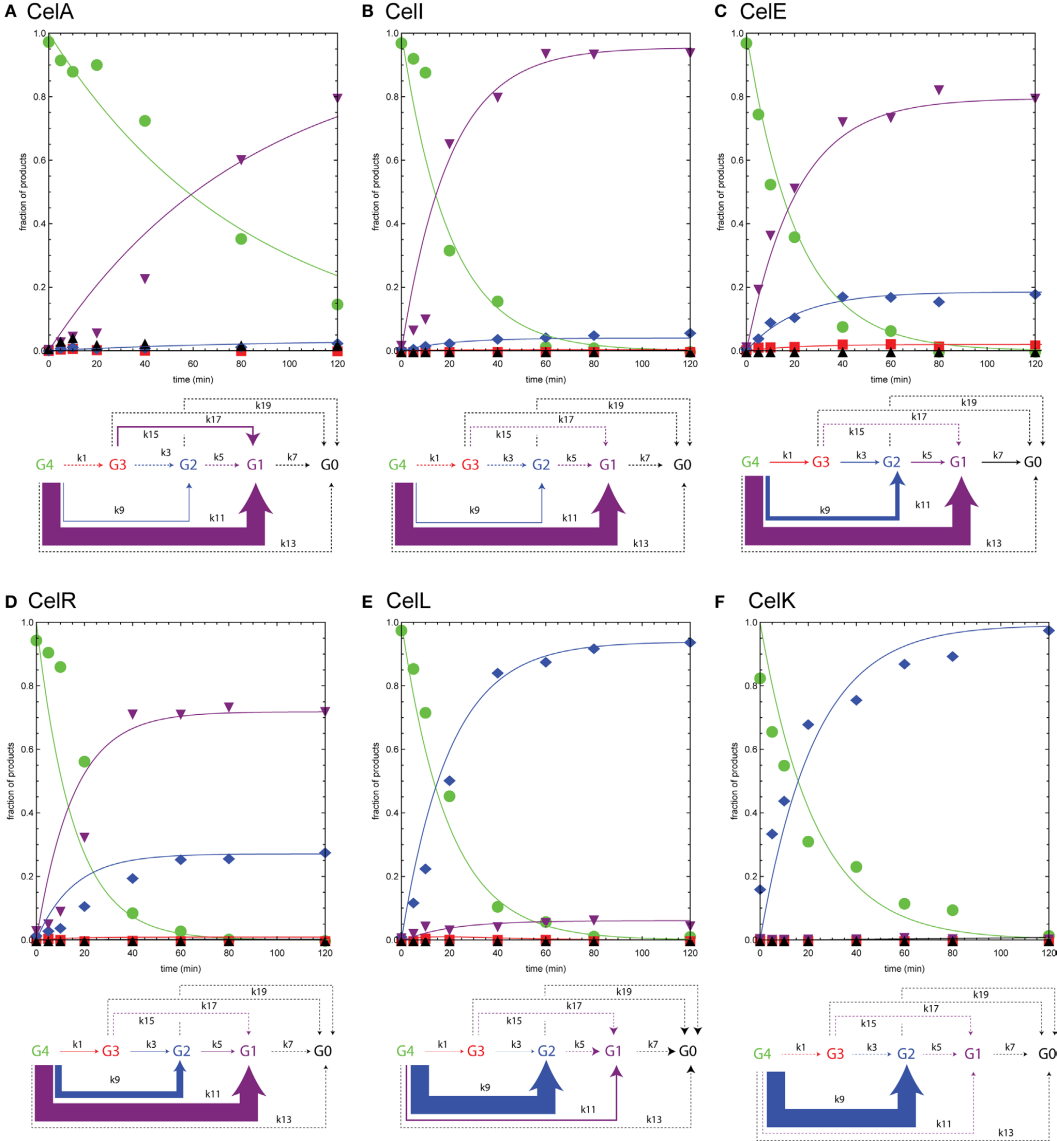
**Numerical analyses of the time courses for reaction of various endoglucanases and cellobiohydrolase with cellotetraose-NIMS**. Products are cellotetraose-NIMS (green), cellotriose-NIMS (red), cellobiose-NIMS (blue), glucose-NIMS (purple), and aglycone-NIMS (black). Relative magnitudes of the apparent rates shown in Table 2 indicated by width of arrows in the modified kinetic scheme. A dashed line indicates that the apparent rate was zero. **(A)** CelA; **(B)** Ce6lI; **(C)** CelE; **(D)** CelR; **(E)** CelL; and **(F)** CelK.

Figure 5 shows time courses for reactions of endoglucanases CelA, CelI, CelE, CelR, CelL, and cellobiohydrolase CelK with cellotetraose-NIMS. The reactions of the individual enzymes were carried out and evaluated as described above for Figure 4. The appearance of the reaction time courses and the relative rates observed are markedly different than observed for BglA. Unlike the β-glucosidase reaction, no intermediates were observed to form and decay, and the central reactions corresponding to release of glucose units were negligible. This seemingly corresponds with the requirement of endoglucanases for a longer oligosaccharide chain to occupy the active site as a determinant of productive binding and catalysis. In effect, the endoglucanases and cellobiohydrolase primarily reacted only once with the cellotetraose-NIMS probe, leading to a markedly simpler cascade of products than observed for the beta-glucosidase. None of the enzymes characterized in Figure 5 was able to carry out reactions that yielded the aglycone-NIMS product (black up triangles), suggesting unproductive binding or blocking steric interactions of the NIMS product with adjacent features of the active site. In contrast, the β-glucosidase BglA (Figure 4) was able to successively remove all glucose groups from cellotetraose-NIMS to yield aglycone-NIMS.

### Endoglucanase CelA Reactions

CelA (Cthe_0269) is a GH8 endoglucanase. It is one of the most abundantly transcribed and secreted proteins in *C. thermocellum* during growth on cellulosic substrates (Brown et al., 2007; Gold and Martin, 2007; Raman et al., 2011; Riederer et al., 2011). Analysis of the crystal structure of the enzyme suggested that the substrate binding channel was optimally configured to bind a cellopentaose molecule (Alzari et al., 1996).

The functional characterizations of Figure 5 demonstrate a progression in reaction selectivity among the enzymes studied. This is a unique power arising from the combination of time-dependent NIMS with numerical analysis. For CelA (Figure 5A), k11 governed removal of cellotriose from cellotetraose-NIMS, leading to the predominant accumulation of glucose-NIMS (88%, purple down triangles). The alternative removal of cellotriose via the two step pathway of k1 (removal of glucose) and k15 (removal of cellobiose) contributed ~9% to the overall product yields, while reaction via k9 (removal of cellobiose) added only ~3% of total products as cellobiose-NIMS (blue diamonds). It is worth noting that CelA gave the slowest hydrolysis of cellotetraose-NIMS of all enzymes tested, which is reflected in the values of apparent rates reported in Table 2 and also in the shape of the plots in Figure 5. This may also reflect a partial rate diminution caused by a mismatch between cellotetraose-NIMS and a preferred cellopentaose occupying the active site channel.

In our earlier reactions of CelA with IL-SG (Deng et al., 2014), a mixture of glucose, cellobiose, triose, and tetraose was observed. Other than cellotetraose, whose release from cellotetraose-NIMS was probably prevented by improper binding of the NIMS moiety in the active site channel, the suite of products given by CelA reaction with cellotetraose-NIMS was comparable to that observed from reactions with the pretreated biomass (Deng et al., 2014).

### Endoglucanase CelI, CelE, and CelR Reactions

For the reactions of CelI (Figure 5B), CelE (Figure 5C), and CelR (Figure 5D), the dominant pattern of preferred removal of cellotriose units to yield glucose-NIMS (purple down triangles) was retained. However, functional differences of these three enzymes were identified as the removal of cellobiose leading to cellobiose-NIMS (blue diamonds) assumed an increasing contribution to the total product distribution. For example, the observed change corresponds to an approximately eightfold increase in k9 between CelI and CelR. In the middle of these boundary enzymes, CelE was unique among the endoglucanases tested as it was also able to release a glucose unit from cellotetraose-NIMS in ~2% yield. In reactions with IL-SG and ammonia fiber expansion pretreated switchgrass (AFEX-SG) (Deng et al., 2014), these three enzymes released a mixture of glucose, cellobiose, and cellotriose, with the distribution of products in the biomass reaction shifted toward cellobiose and glucose. However, this shift is, in part, due to the ability of these enzymes to cleave solubilized cellotriose into cellobiose and glucose. Subsequent hydrolysis of released oligosaccharides could not be detected when cellotetraose-NIMS was the substrate.

CelI (Cthe_0040) has a structure consisting of GH9 and two CBM3 domains (Hazlewood et al., 1993). It catalyzes the hydrolysis of 1,4-β-glucosidic linkages in cellulose and other glucans. The structure suggests the position of a tunnel that can permit the release of either cellotriose or cellobiose from cellotetraose-NIMS (PDB 2XFG, no associated publication).

CelE (Cthe_0797) is a multidomain enzyme consisting of GH5, dockerin, and GSDL-lipase domains. Our work has shown that the GH5 domain has broad specificity for reaction with cellulose, xylan, mannan, xyloglucan, and other polysaccharides (Deng et al., 2014; Takasuka et al., 2014). The active site channel of this enzyme is open and tolerates the placement of each of these different linear and branched polysaccharides in a way that a glycosidic bond can be placed in the appropriate position for hydrolysis (Bianchetti et al., 2015). The release of cellotriose, cellobiose, and glucose from cellotetraose-NIMS is compatible with this broad specificity active site. Nevertheless, the active site is not sufficiently tolerant to remove cellotetraose, leading to the formation of aglycone-NIMS.

Previous studies have reported that CelR (Cthe_0578) is a β-glucanase with preference for release of cellotetraose in reactions with amorphous cellulose (Zverlov et al., 2005). Subsequently, CelR was able to convert the longer solubilized oligosaccharide to shorter oligosaccharides. The present studies provide support for this conclusion, as k11 for release of cellotriose was the predominant reaction with cellotetraose-NIMS. Our studies of CelR in reactions with IL-SG and AFEX-SG gave glucose and cellobiose as the dominant hydrolysis products (Deng et al., 2014), suggesting a kinetically rapid conversion of longer oligosaccharides to shorter during the duration of the reaction. Removal of cellotetraose was not observed from cellotetraose-NIMS, which as proposed above likely represents ineffective binding of the NIMS probe in the active site adjacent to the active site.

### Endoglucanase CelL and Cellobiohydrolase CelK Reactions

We tested the cellotetraose-NIMS reactions with an additional endoglucanase, CelL (Cthe_0405, Figure 5E), and a reducing end cellobiohydrolase, CelK (Cthe_0212, Figure 5F). These enzymes show a shift in reaction specificity so that removal of cellobiose to produce cellobiose-NIMS (blue diamonds) became the dominant pattern of reaction. Notably, CelL had an approximately threefold enhanced ability to remove cellobiose relative to CelR because of a higher k9 value and also an ~10-fold decrease in the ability to remove cellotriose associated with a lower k11 value (Table 2). CelL reacted with IL-SG also showed preference for release of cellobiose (Deng et al., 2014). Furthermore, although CelK also had an approximately threefold enhanced ability to remove cellobiose relative to CelR because of a higher k9 value, it showed no ability to produce either cellotriose or glucose (e.g., k1 and k11 = 0; Table 2).

The high specificity for release of cellobiose by a cellobiohydrolase is a characteristic reactivity (Amano et al., 1996; Barr et al., 1996; Divne et al., 1998), including CelK (Kataeva et al., 1999) and also CelK reacted with IL-SG (Deng et al., 2014). Thus, cellotetraose-NIMS clearly reports on this catalytic function of CelK. There are no previously published reactivity studies or crystal structures of CelL, beyond our studies of reaction with IL-SG, where CelL showed strong preference for release of cellobiose and xylobiose from the pretreated biomass (Deng et al., 2014).

## Conclusion

This work establishes the utility of a chemically synthesized mass spectral probe for characterization of GHs. We have shown remarkable correspondence between the products obtained from enzyme reactions with the synthetic cellotetraose-NIMS probe and IL- and AFEX-pretreated switchgrass (Deng et al., 2014). Because of the emerging success of robotic cell-free translation to provide active enzyme samples from synthesized genes (Takasuka et al., 2014; Bianchetti et al., 2015), the substantial advantages of automation and miniaturization afforded by the Nimzyme platform (Deng et al., 2012, 2014; de Rond et al., 2013; Heins et al., 2014), and the predictive power inherent in numerical analysis of enzyme reaction time courses (Cleland, 1975; Orsi and Tipton, 1979; Duggleby, 1995; Marangoni, 2003), our combination offers a powerful new approach for functional annotation of bioenergy phylogenetic space.

## Author Contributions

KD, TT, CB, LB, PA, TN, and BF designed experiments, carried out experimental work, analyzed results, and prepared the manuscript. All authors read and approved the final manuscript.

## Conflict of Interest Statement

Kai Deng and Trent R. Northen are coinventors on a patent application that covers the oxime-NIMS assay. Taichi E. Takasuka, Christopher M. Bianchetti, and Brian G. Fox are coinventors on a patent application that covers use of multifunctional enzymes. Lai F. Bergeman and Paul D. Adams have no conflict of interest to declare.

## Abbreviations

AFEX-SG, ammonia fiber expansion pretreated switchgrass; CBM, carbohydrate-binding module; CelE, broad specificity GH family 5 (GH5) domain from *C. thermocellum* Cthe_0797; GH, glycoside hydrolase; IL-SG, ionic liquid pretreated switchgrass; NIMS, nanostructure-initiator mass spectrometry.
